# Dexamethasone Inhibits Repair of Human Airway Epithelial Cells Mediated by Glucocorticoid-Induced Leucine Zipper (GILZ)

**DOI:** 10.1371/journal.pone.0060705

**Published:** 2013-04-03

**Authors:** Jingyue Liu, Mingxiang Zhang, Chao Niu, Zhengxiu Luo, Jihong Dai, Lijia Wang, Enmei Liu, Zhou Fu

**Affiliations:** 1 Department of Respiratory Medicine, Children's Hospital of Chongqing Medical University, Chongqing, China; 2 Respiratory Research Laboratory, Ministry of Education Key Laboratory of Child Development and Disorders, Children's Hospital of Chongqing Medical University, Chongqing, China; University of Washington, United States of America

## Abstract

**Background:**

Glucocorticoids (GCs) are a first-line treatment for asthma for their anti-inflammatory effects, but they also hinder the repair of airway epithelial injury. The anti-inflammatory protein GC-induced leucine zipper (GILZ) is reported to inhibit the activation of the mitogen-activated protein kinase (MAPK)-extracellular-signal-regulated kinase (ERK) signaling pathway, which promotes the repair of airway epithelial cells around the damaged areas. We investigated whether the inhibition of airway epithelial repair imposed by the GC dexamethasone (DEX) is mediated by GILZ.

**Methods:**

We tested the effect of DEX on the expressions of *GILZ* mRNA and GILZ protein and the MAPK-ERK signaling pathway in human airway epithelial cells, via RT-PCR and Western blot. We further evaluated the role of GILZ in mediating the effect of DEX on the MAPK-ERK signaling pathway and in airway epithelium repair by utilizing small-interfering RNAs, MTT, CFSE labeling, wound-healing and cell migration assays.

**Results:**

DEX increased *GILZ* mRNA and GILZ protein levels in a human airway epithelial cell line. Furthermore, DEX inhibited the phosphorylation of Raf-1, Mek1/2, Erk1/2 (components of the MAPK-ERK signaling pathway), proliferation and migration. However, the inhibitory effect of DEX was mitigated in cells when the *GILZ* gene was silenced.

**Conclusions:**

The inhibition of epithelial injury repair by DEX is mediated in part by activation of GILZ, which suppressed activation of the MAPK-ERK signaling pathway, proliferation and migration. Our study implicates the involvement of DEX in this process, and furthers our understanding of the dual role of GCs.

## Introduction

Asthma is a chronic inflammatory airway disorder accompanied by airway epithelial cell damage. The airway epithelium acts as a barrier between the internal and external environment, and has a crucial role in maintaining normal airway structure and function, from the trachea to the alveoli. Thus, the airway epithelium is the first to contact inhaled allergens, physical stimuli, pollution, viruses, bacteria, and respiratory drugs [1]. If the airway epithelium undergoes prolonged and repeated damage, and there is no appropriate repair process, the integrity of the airway is destroyed and repair is further delayed. There is evidence that in asthma the repair process is fundamentally flawed, and is associated with activation of the epithelial-mesenchymal trophic unit and growth factors that cause pathological airway remodeling [2]. Studies have also revealed that patients with asthma have abnormal airway epithelial shedding, and almost all asthma patients show on endobronchial biopsy a variable degree of airway epithelial damage [3], [4].

Inhaled glucocorticoids (GCs) have anti-allergy, anti-inflammatory, and immunosuppressive properties, in addition to regulating the biosynthesis and metabolism of key nutrients such as sugars, fats, and proteins. GCs have been widely used in the treatment of asthma, rheumatoid arthritis, and chronic obstructive pulmonary disease, among other disorders [5], [6]. As one of the most effective medications to prevent and treat asthma, GCs primarily act on airway epithelium to inhibit airway inflammation. However, studies have shown that GCs also adversely affect the repair process by suppressing early-stage migration and proliferation of airway epithelial cells [7], [8]. The molecular mechanisms underlying the dual effects of GCs in these processes remain unclear.

Glucocorticoid-induced leucine zipper (*GILZ*) was first described in 1997 as a dexamethasone (DEX)-responsive gene [9]. GILZ is a member of the TSC-22 (transforming growth factorβ-stimulated clone 22) family. Members of this family are widely expressed and they affect multiple biological processes. These molecules contain three domains: a high degree of homology in the dimerization domain (TSC-22 box and LZ pattern), and different N-terminal and C-terminal domains [10]. GILZ is expressed in the liver, kidney, lung, and brain as well as other tissues or cells. GCs, ethanol, interleukin (IL)10, and even certain bacterial species such as *Yersinia enterocolitica* and *Clostridium difficile* further induce the expression of GILZ [11]–[15]. GILZ has been reported to be involved in cellular proliferation and apoptosis, control of T-cell activation and development, modulation of IL2 production, and increase of epithelial sodium channel-mediated sodium transport [16]–[19].

GILZ is involved in GC-induced anti-inflammatory and immunosuppressive responses [20]; it inhibits the activation of transcription factors, including nuclear factor-kappaB (NF-κB) and activator protein 1 (AP-1) [21], [22]. GILZ also inhibits the mitogen-activated protein kinase (MAPK)-extracellular-signal-regulated kinase (ERK) signaling pathway, by binding directly to the upstream regulator V-raf-1 murine leukemia viral oncogene homolog 1 (RAF1) to prevent phosphorylation [23]. The MAPK-ERK signaling pathway is involved in airway epithelial repair after injury and promotes the proliferation and migration of neighboring cells around the injury site [7], [8], [24].

In the present study we determined whether DEX induces the expression of GILZ in human airway epithelial cells, using the cell line 9HTE. We investigated whether the inhibition of airway epithelial repair imposed by the GC dexamethasone (DEX) is mediated by GILZ, and associated changes in the MAPK-ERK signaling pathway and cellular proliferation and migration.

## Materials and Methods

### Cell culture

Cells of the cell line 9HTE (a Simian virus 40 [SV40]-immortalized line of human tracheal epithelial cells) [25], provided by Respiratory Research Laboratory, Ministry of Education Key Laboratory of Child Development and Disorders, Children's Hospital, Chongqing, China, were grown in Dulbecco's modified Eagle's medium (DMEM) supplemented with 10% fetal bovine serum (FBS; Gibco, USA). The cells were incubated at 37°C in a 5% CO_2_ atmosphere, and growth status was observed under an inverted microscope. Upon reaching 80%–90% confluence, cells were digested with trypsin and subcultured.

### Reverse transcription PCR (RT-PCR)

9HTE cells (5×10^5^/well) were cultured in 6-well plates and stimulated with control or 10 µM dexamethasone (DEX; D1756, Sigma, USA) for up to 24 h. Total RNA was then extracted from the 9HTE cells using lysis buffer (Bioteke, Beijing, China) and reversed transcribed with a PrimeScriptRT reagent kit (TaKaRa, Shiga, Japan) as suggested by the manufacturers. The mRNA expression level of *GILZ* was measured by semiquantitative reverse transcription PCR (RT-PCR). Two microliters of cDNA were amplified in a total of 25 µL volume reaction, containing 12.5 µL 2× Master Mix, 0.5 µL each of sense and antisense primers (10 µM), and 9.5 µL ddH_2_O. The sequences of the primers were *GILZ* forward: 5′-TGGTGGTTCTGCGGTGTAAGTG-3′, reverse: 5′-CTCCTCGTGAGATGATGCTTGG-3′; β-actin forward: 5′-GTGGACATCCGCAAAGAC-3′, reverse: 5′-GAAAGGGTGTAACGCAACT-3′. The amplification of *GILZ* was performed at 94°C for 4 min; followed by 35 cycles of 94°C for 30 s, 64°C for 30 s, 72°C for 45 s; and then an extension at 72°C for 5 min. The expected product size was 115 bp. The β-actin conditions for amplification were: 94°C for 4 min; 30 cycles of 94°C for 30 s, 60°C for 30 s, 72°C for 45 s, and an extension at 72°C for 5 min. The expected product size was 303 bp. PCR products were resolved by electrophoresis in 1.5% agarose gels and visualized with ethidium bromide staining. The ratio of the PCR products *GILZ*-to-β-actin was measured by densitometry. β-actin was used as an internal reference.

### Real-time RT-PCR

The preparation of total RNA and cDNA was obtained as described above. The reaction was performed using RealMasterMix (SYBR Green; Tiangen, Beijing, China) in a total 20 µL volume containing 2 µL cDNA, 9 µL SYBR solution, 0.5 µL each of sense and antisense primers (10 µM), and up to 8 µL ddH_2_O. The primer sequences of *GILZ* were as above, and the primer sequences for the housekeeping gene glyceraldehyde 3 phosphate dehydrogenase (*GADPH*) were forward: 5′-AAGAAGGTGGTGAAGCAGGC-3′ and reverse: 5′-TCCACCACCCTGTTGCTGTA-3′. The PCR amplification conditions for *GAPDH* were 95°C for 3 min; and 40 cycles of 95°C for 10 s and 60°C for 30 s. The expected product size was 203 bp. The amplification conditions for *GILZ* were 95°C for 3 min, then 40 cycles of 95°C for 10 s, and 64°C for 30 s.

### Cell transfection

9HTE cells were seeded in culture plates in serum-free DMEM without antibiotics. Three small-interfering RNAs (siRNAs) were designed and synthesized targeting *GILZ* (*GILZ*_1−3_ siRNAs), as well as a non-specific si-RNA (si-negative control), and a *GAPDH* si-RNA (si-positive control) (Benefit and Invitrogen, Shanghai, China). These were transfected into separate groups of 9HTE cells using Lipofectamine 2000 (Invitrogen, Carlsbad, CA, USA) in accordance with the manufacturer's instructions. The *GILZ*_1–3_ siRNAs were evaluated for the most effective *GILZ* si-RNA. The sequences of the three *GILZ* siRNAs were *GILZ*_1_ si-RNA: 5′-GGAUCUGGUGAAGAAUCAUTT-3′, *GILZ*_2_ si-RNA: 5′-GAACUCCCAGCUAGAGCGUTT-3′, and *GILZ*_3_ si-RNA: 5′-GUUCCAGUCCUGUCUGAGCTT-3′. After 6 h of transfection, fresh medium was added to the transfected cells to culture for up to 24 h, and were then stimulated with DEX (10 µM) for an additional 24 h. The cells were collected to verify gene silence by determining the expression of *GILZ* mRNA and GILZ protein using real-time PCR and Western blot, respectively.

### Cyto-immunofluorescence staining

9HTE cells (5×10^4^ cells/well) were cultured in 24-well plates, in which the glass slides were placed in the bottom. The treated cells were fixed with 4% paraformaldehyde for 30 min. After washing with phosphate-buffered saline (PBS), slides were incubated with 0.1% TritonX-100 for 10 min to penetrate the membrane, and blocked with 5% bovine serum albumin for 30 min at room temperature. The slides were incubated with GILZ monoclonal antibody (Santa Cruz Bio, CA, USA) at 1∶50 dilution overnight at 4°C, followed by incubating with DyLight^™^ 488 conjugated secondary antibody at 37°C for 45 min. Finally the slides were washed and dyed with 4′,6-diamidino-2-phenylindole (DAPI), mounted, and examined by fluorescence microscopy. The negative control was incubated with PBS instead of GILZ antibody.

### Western blot

The protein from 9HTE cells was collected and protein concentration was determined by bicinchoninic acid assay. Equal amounts of protein from whole cell lysates were solubilized in 5× sodium dodecyl sulfate (SDS)-sample buffer and separated on 10–15% SDS polyacrylamide gels (KeyGEN, Nanjing, China) according to molecular weight. Separated proteins were transferred onto a polyvinylidene fluoride membrane. After blocking, the membrane was incubated with anti-GILZ antibody (1∶100 dilution, Santa Cruz Bio, CA, USA)and sequentially with anti-Raf-1, p-Raf-1, Mek1/2, p-Mek1/2, Erk1/2, p-Erk1/2 antibodies (1∶1000 dilution, Cell Signaling, Danvers, MA, USA) overnight at 4°C. Afterwards, horseradish peroxidase-conjugated anti-mouse/rabbit secondary antibodies (MultiSciences, Hangzhou, China) were used. Blots were visualized using an enhanced chemiluminescence kit (KeyGEN, Nanjing, China).

### MTT assay

Cell proliferation was analyzed using a methyl-thiazolyl-tetrazolium (MTT) bromide assay (Amresco, USA). Briefly, the cells were seeded in 96-well plates at a density of 10 000 cells/well, and transfected with non-specific si-RNA or *GILZ* si-RNA for up to 48 h. Afterwards 10 µL MTT (5 mg/mL) was added and incubated for an additional 4 h. The supernatants were removed and 150 µL dimethyl sulfoxide (Amresco, USA) was added to each well. There were five duplicate wells in each group and the experiment was repeated three times. The absorbance value (optical density) of each well was measured at 490 nm.

### CFSE (5(6)-carboxyfluorescein diacetate succinimidyl ester) labeling

9HTE cells were suspended in pre-warmed PBS at a final concentration of 1×10^6^ cells/mL, and 5 µM CFSE (Invitrogen, Carlsbad, CA, USA) was added to the cell suspension, and the cells were incubated at 37°C for 10 min. The labeling process was quenched by the addition of 10 volumes of ice-cold culture medium (10% FBS), and the cell suspension was incubated 5 min on ice and subsequently centrifuged. CFSE-labeled cells were washed twice with medium, and were seeded in 24-well plates at a density of 1×10^5^ cells per well. Cultured cells from each well were harvested after transfection and treated with DEX, and the CFSE fluorescence intensity was measured by flow cytometry.

### Wound-healing assay

9HTE cells were seeded in 24-well plates at a density of 1×10^5^ cells per well. After the cells were transfected with non-specific si-RNA or *GILZ* si-RNA for 24 h and reached approximately 80%-90% confluence, the cells were scratched with a 10 µL pipette tip and cultured in the presence of 10 µM DEX for an additional 24 h. Images of the wound were recorded using a fluorescence microscope, immediately after wounding (0 h) and after culturing (24 h). The cells of both sides around the damaged area migrated toward the cell-free area. The wound widths of three different wound surfaces in each group were noted and subsequently measured using image J analysis software. The experiment was repeated three times.

### Cell migration assay

9HTE cells were transfected with non-specific si-RNA or *GILZ* si-RNA for 24 h, followed by digesting, counting, and suspension in serum-free DMEM. One hundred-microliter cell suspensions (2×10^5^cells) were seeded in the upper chamber of a transwell unit with an 8.0 µm polycarbonate membrane (Millipore, Boston, USA) inserted in a 24-well plate, and 600 µL culture medium with 10% FBS was placed in the lower chamber where DEX (10 µM) was added. After the cells were incubated for 24 h at 37°C, the cells on the top surface of the transwell chamber were removed with a cotton swab. The cells adhering to the lower surface were fixed with 4% paraformaldehyde for 30 min, stained with hematoxylin, and counted under a microscope in five randomly chosen fields.

### Statistical analysis

Experimental differences were assessed for statistical significance using analysis of variance (ANOVA) and SPSS 16.0 software. Data were expressed as mean±standard deviation and *P*-values <0.05 were considered significant (*P*<0.05).

## Results

### Expression of *GILZ* mRNA and GILZ protein in 9HTE cells

**Figure 1:**
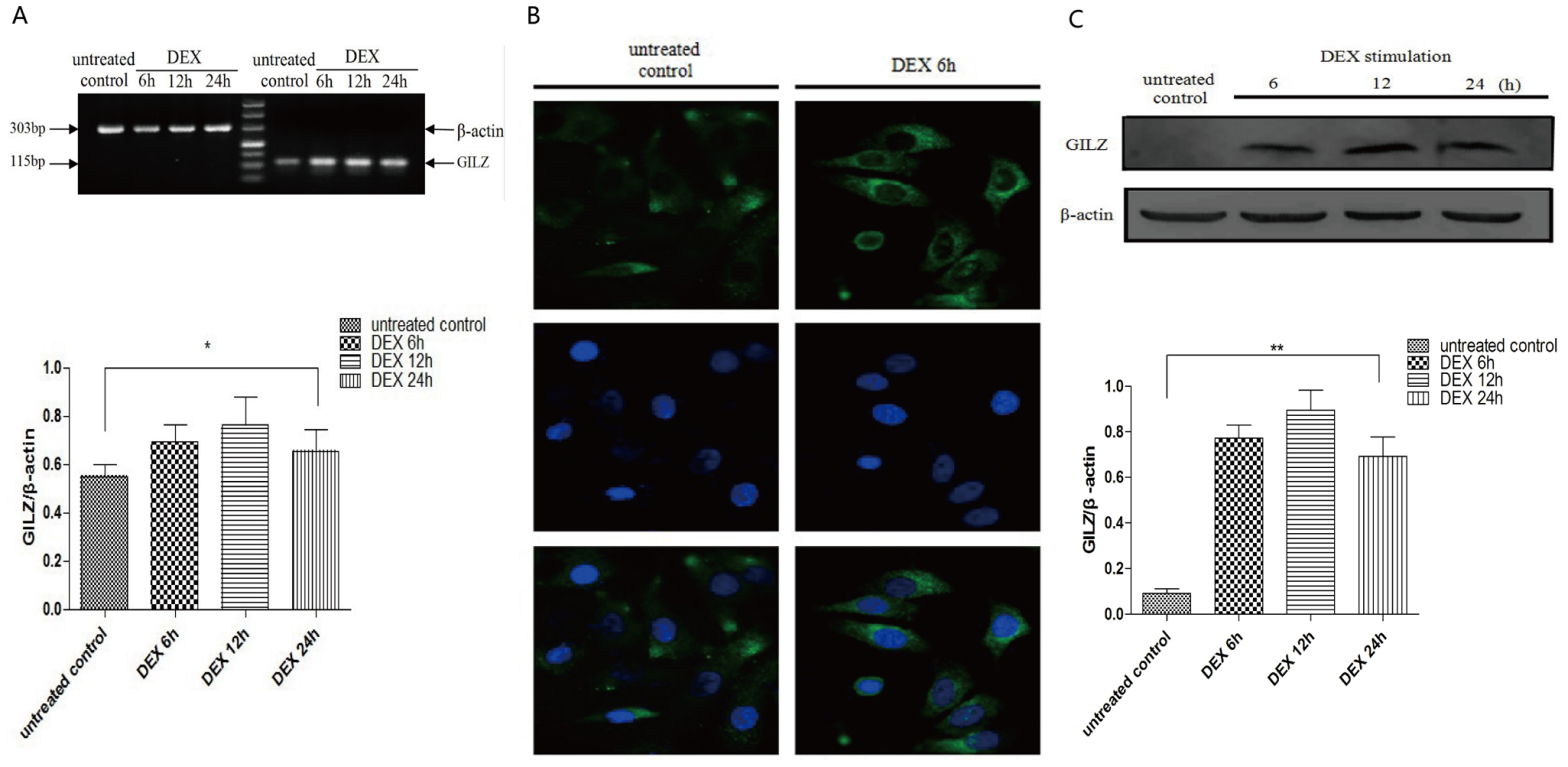
The effect of DEX on *GILZ* mRNA and GILZ protein expression in 9HTE cells. (A) RT-PCR was performed to detect the *GILZ* mRNA level and showed that DEX induced the expression of *GILZ* mRNA starting from 6 h until 24 h (for 24 h, 0.553±0.048 in the untreated group compared with 0.658±0.087 in the DEX-treated, *P<0.05, n = 8). (B) 9HTE cells were treated with or without DEX for 6 h, and GILZ was located using immunofluorescence (400×). (C) Western blot was performed to determine the expression levels of GILZ protein from 6 h until 24 h with DEX (for 24 h, 0.092±0.019 in the untreated group compared with 0.693±0.085 in the DEX-treated, **P<0.001, n = 3). β-actin was used as the loading control.

We tested the effect of DEX on the expression of *GILZ* mRNA and GILZ protein in cells of the SV40-immortalized line of human tracheal epithelial cells 9HTE. We found that DEX (10 µM) significantly induced mRNA levels of *GILZ* starting at 6 h, and levels continued to increase 24 h after treatment compared to the untreated control cells (*P* <0.05), as determined by RT-PCR (Figure 1A). The results from the immunofluorescence assays showed that GILZ protein was mainly located in the cytoplasm, and DEX significantly increased the expression of GILZ protein at 6 h compared with the untreated control group (Figure 1B). Similar results showing increased GILZ protein levels were also found via Western blot analysis (Figure 1C). However, no further induction was observed at longer treatment times. Thus, GILZ was capable of being induced rapidly and obviously by DEX in airway epithelial cells *in vitro*.

### Identification of the most effective GILZ si-RNA

**Figure 2:**
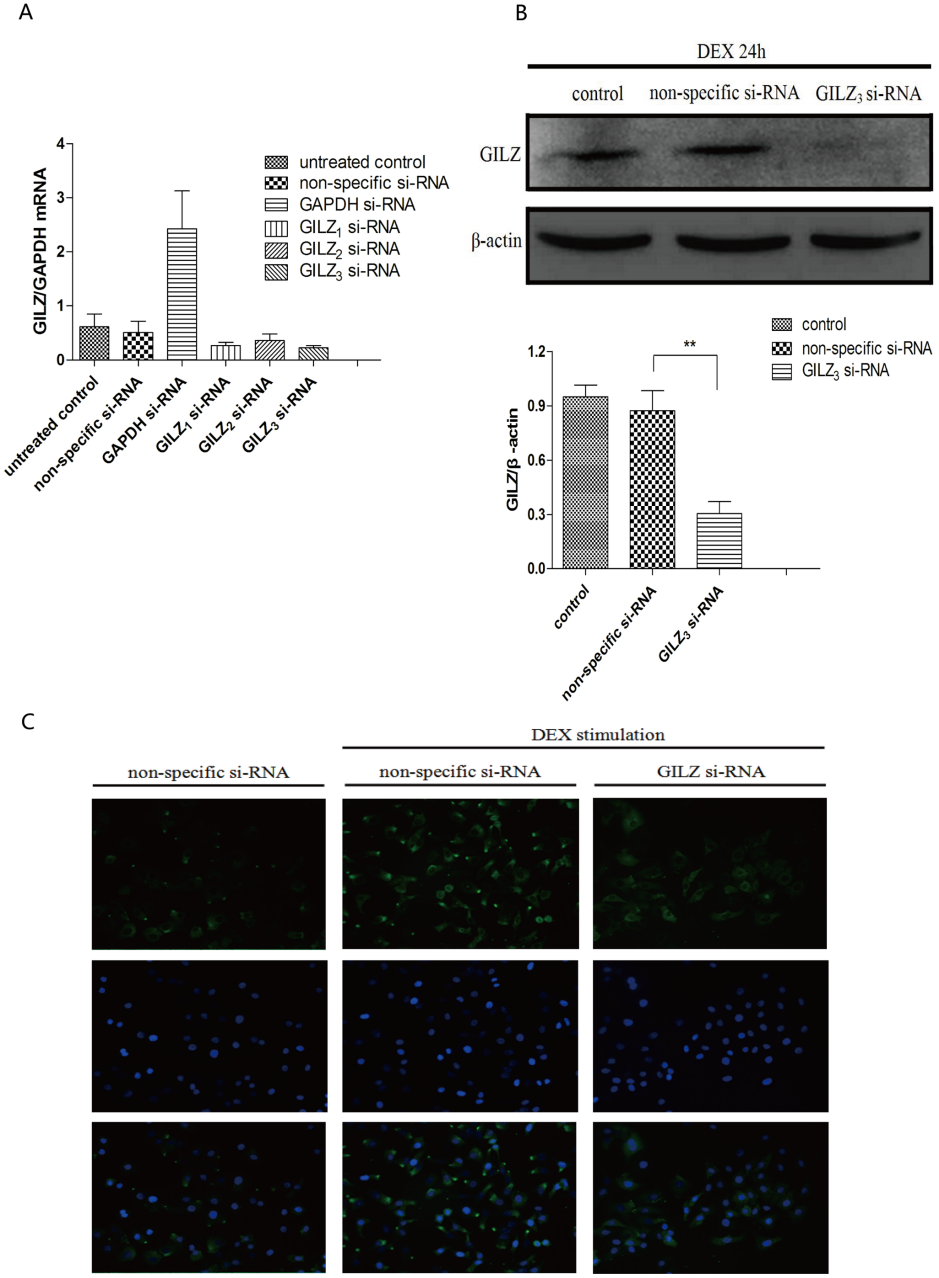
GILZ expression in 9HTE cells transfected with *GILZ* siRNAs. (A) Real time-PCR was performed to show transcriptional levels of the *GILZ* gene 48 h after transfection with *GILZ*_1−3_ siRNAs of cells treated with DEX for 24 h. Non-specific si-RNA was the negative control, and *GAPDH* si-RNA was the positive control (n = 3). (B) Cellular GILZ protein was collected from 9HTE cells transfected with non-specific si-RNA or *GILZ*_3_ si-RNA for 48 h. Afterwards, GILZ protein levels were detected by Western blot (0.873±0.109 in the non-specific si-RNA group compared with 0.305±0.065 in the *GILZ*_3_ si-RNA group, **P<0.001, n = 3), β-actin was used as a loading control. (C) The expression of GILZ protein was detected by immunofluorescence (200×) after 9HTE cells were transfected with non-specific si-RNA or *GILZ* si-RNA, separately for 48 h.

Three *GILZ* siRNAs (*GILZ*_1−3_ siRNAs) that targeted different mRNA sequences were transfected into 9HTE cells. We determined the relative silencing efficiency of these siRNAs by real-time PCR to select the best for subsequent experiments (Figure 2A). There was no statistical difference between the ratios of *GILZ*-to-*GAPDH* in the untreated control group and non-specific group (*P* >0.05). However, the ratio of *GILZ*-to-*GAPDH* in the *GILZ*_1_-, *GILZ*_2_-, and *GILZ*_3_ si-RNA groups were 0.27±0.06, 0.36±0.12, and 0.22±0.04, respectively. These results revealed that *GILZ*_3_ si-RNA had the highest silencing efficiency, and the expression of *GILZ* in the group transfected with *GILZ*_3_ si-RNA was 55.8% that of the non-specific si-RNA group.

Western blot was then used to detect the GILZ protein expression in 9HTE cells transfected with *GILZ*_3_ si-RNA. We found that *GILZ*_3_ si-RNA significantly silenced GILZ protein expression (*P* <0.001, compared with the non-specific si-RNA group), and there was no statistical difference between the control and non-specific groups (Figure 2B). We further confirmed the expression of GILZ protein by immunofluorescence, which was significantly decreased in cells where *GILZ* gene was silenced by the si-RNA (Figure 2C). Therefore, *GILZ*_3_ si-RNA was used for subsequent experiments.

### Increased expression of GILZ induced by DEX inhibited activation of the MAPK-ERK signaling pathway

**Figure 3:**
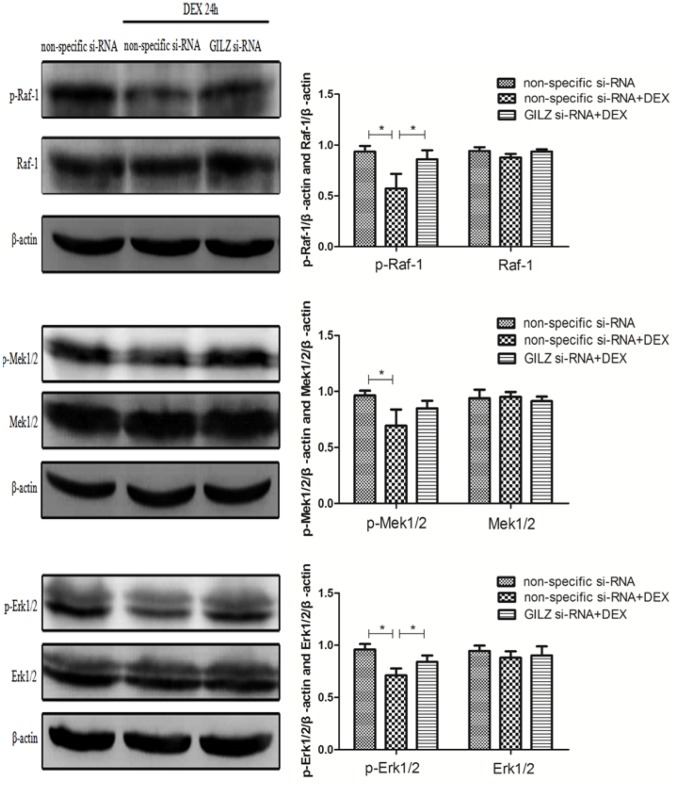
Differential expressions of components of the MAPK-ERK pathway in non-specific si-RNA, DEX-treated/non-specific si-RNA, and DEX-treated/*GILZ* si-RNA-transfected 9HTE cells. Cellular proteins were collected from 9HTE cells transfected with non-specific si-RNA or *GILZ* si-RNA in the absence or presence of DEX for 24 h. Western blot was performed to detect levels of the phosphorylated forms of Raf-1, Mek1/2, and Erk1/2, and the respective total proteins (p-Raf-1, p-Mek1/2, p-Erk1/2: 0.935±0.056, 0.965±0.042, 0.959±0.052 in the non-specific si-RNA group compared with 0.574±0.143, 0.694±0.145, 0.712±0.066 in the DEX-treated/non-specific si-RNA group, compared with 0.861±0.087, 0.849±0.067, 0.840±0.061 in the DEX-treated/*GILZ* si-RNA group, n = 3). *Indicates a significant difference (*P*<0.05), β-actin was used as the loading control.

The MAPK-ERK signaling pathway is a key factor in the regulation of cell survival, apoptosis, proliferation, migration, and differentiation to promote airway epithelial repair [26]; GILZ induced by DEX inhibited the activation of the MAPK-ERK signaling pathway [23]. We investigated via Western blot whether increased DEX-induced GILZ inhibited the activation of several members of the MAPK-ERK pathway. We found that DEX inhibited the phosphorylation of Raf-1, Mek1/2, and Erk1/2 (*P* <0.05, compared with the non-specific si-RNA group), which was not observed in cells in which GILZ expression was silenced (*P* >0.05, compared with the non-specific si-RNA group), and the amount of total Raf-1, Mek1/2, and Erk1/2 proteins was unchanged (Figure 3). These data indicated that expression of GILZ is involved in the activation of the MAPK-ERK signaling pathway.

### GILZ mediated DEX to inhibit proliferation and migration of 9HTE cells

**Figure 4:**
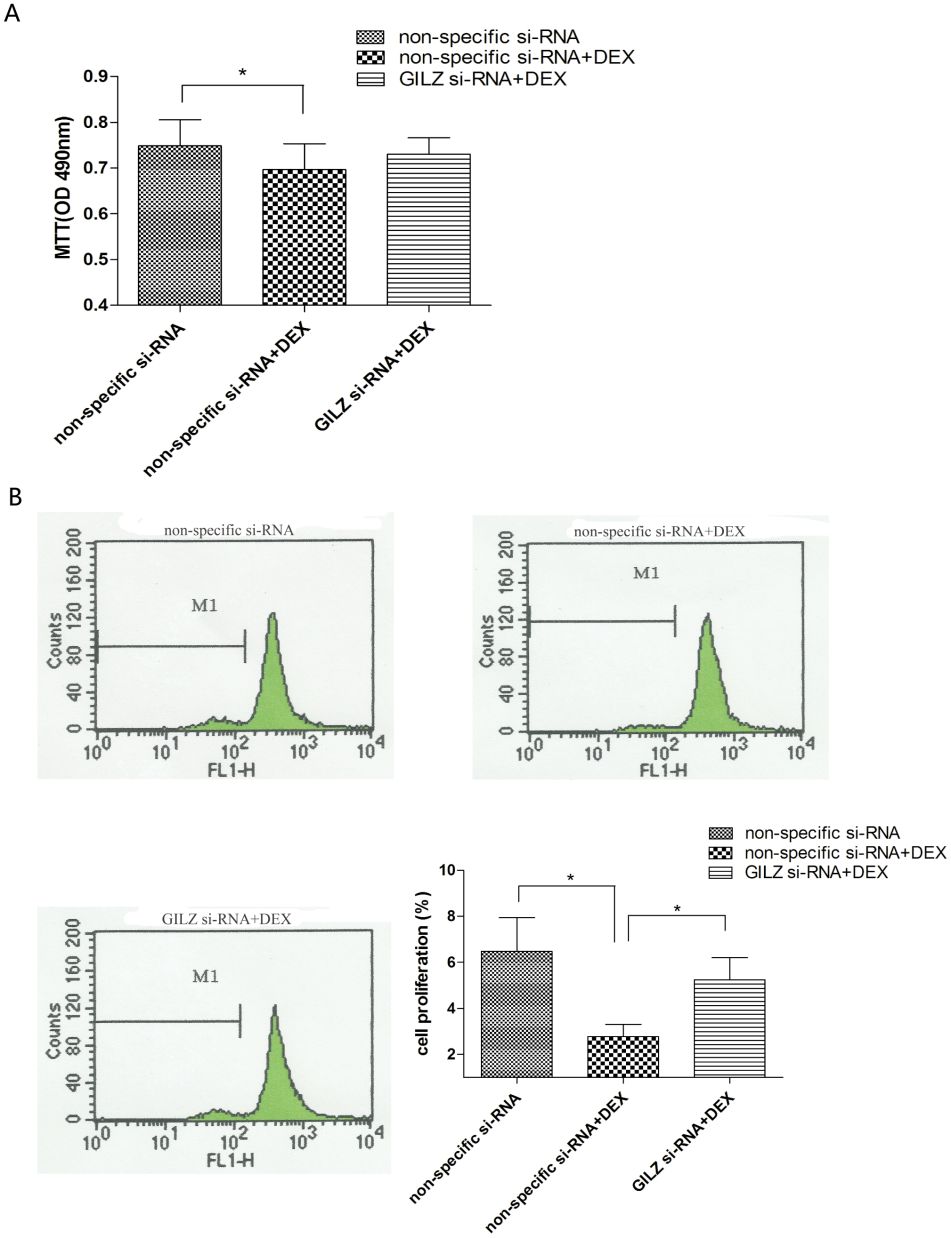
Silencing of *GILZ* partially abrogated the effect of DEX on proliferation of 9HTE cells. (A) 9HTE cells were transfected with non-specific si-RNA or *GILZ* si-RNA in the absence or presence of DEX. The effect of *GILZ* knockout on 9HTE cell proliferation was measured via MTT assay, and absorbance was read at 490 nm (0.749±0.057 in the non-specific si-RNA group compared with 0.697±0.057 in the DEX-treated/non-specific si-RNA group, *P<0.05, n = 3). (B) 9HTE cells were labeling with CFSE and the CFSE fluorescence intensity was measured by flow cytometry (6.468±1.463 in the non-specific si-RNA group and 5.233±0.970 in the DEX-treated/GILZ si-RNA group compared with 2.765±0.539 in the DEX-treated/non-specific si-RNA group, *P<0.05, n = 4).

We examined the effect of DEX on cell proliferation using an MTT assay. We found that DEX inhibited cell proliferation (*P* <0.05, compared with the non-specific si-RNA group), which was overcome by downregulation of GILZ using *GILZ* si-RNA (P >0.05, compared with the non-specific si-RNA group; Figure 4A). We labeled cells using CFSE and found that when treated with DEX, cells exhibited less proliferation compared with those in the non-specific si-RNA group. However, the percentage of cell proliferation was higher in those cells in which the *GILZ* gene was silenced (Figure 4B).

**Figure 5:**
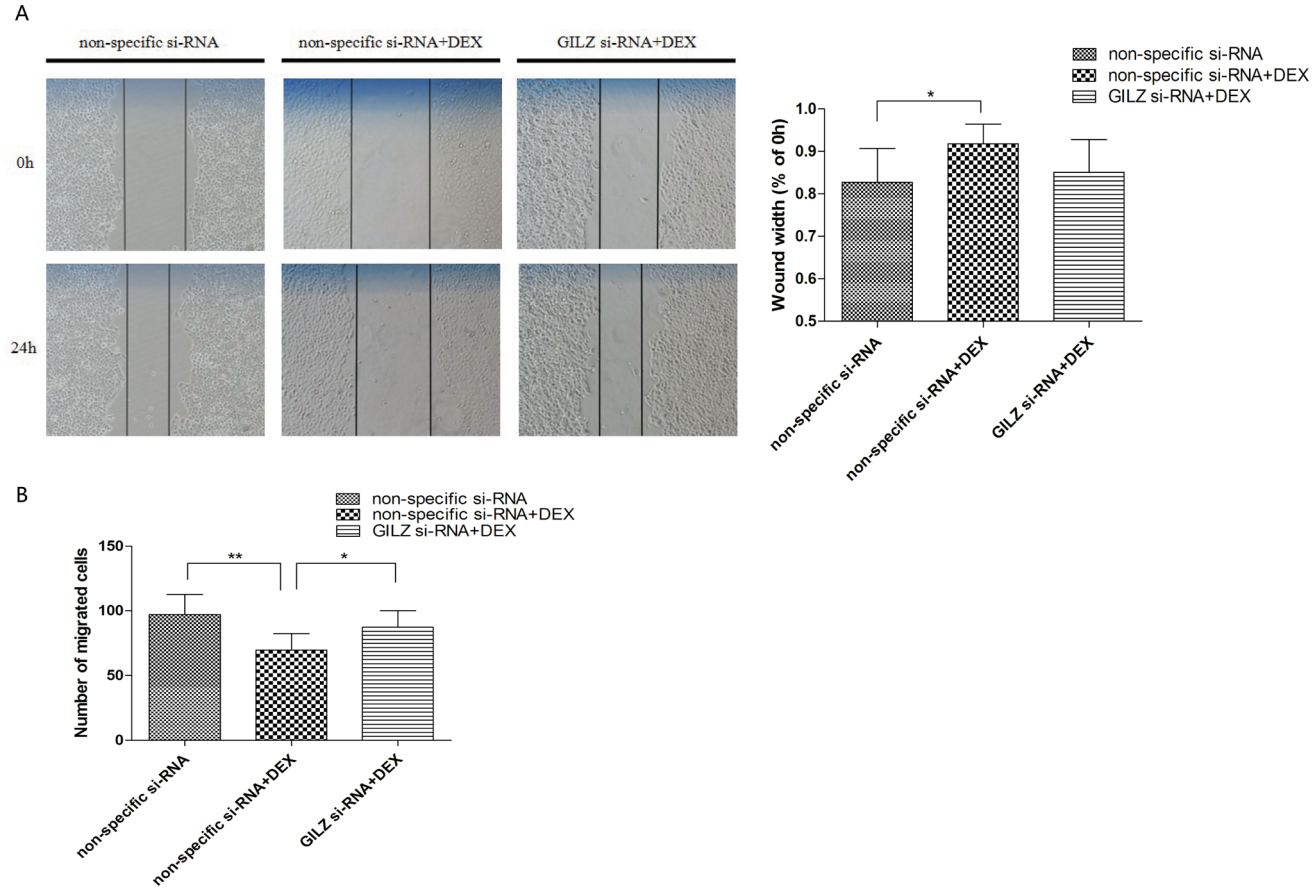
*GILZ* mediated the inhibiting effect of DEX on migration of 9HTE cells. (A) Wound sites (area cleared of cells in the center of the scraped area) were observed and photographed. Photographs showed the repair of the wound in the three groups. (0.827±0.080 in the non-specific si-RNA group compared with 0.918±0.045 in the DEX-treated/non-specific si-RNA group, *P<0.05, n = 3). (B) 9HTE cell migration was examined by transwell chamber and counted under a microscope in five randomly chosen fields of each group, three independent experiments (97.066±15.448 in the non-specific si-RNA group and 87.266±12.876 in the DEX-treated/*GILZ* si-RNA group compared with 69.733±12.572 in the DEX-treated/non-specific si-RNA group, **P<0.001 and *P<0.05, n = 3).

The wound-healing assay showed that 24 h after wounding DEX treatment inhibited wound closure relative to the non-specific si-RNA group (*P* <0.05; Figure 5A), and the wound width decreased in cells where the *GILZ* gene was silenced.

We further detected cell migration using transwell assays. We found that, compared with the non-specific si-RNA group, fewer cells migrated in the DEX group (*P* <0.001; Figure 5B), but migration was recovered in *GILZ* si-RNA-transfected cells. The *GILZ* si-RNA group overcame the inhibitory effect of DEX and there was no statistical difference compared to the non-specific si-RNA group (Figure 5B). The results suggested that DEX, acting through its effect on GILZ, inhibited proliferation and migration of 9HTE cells.

## Discussion

Although GCs are one of the most effective therapies for asthma, *in vitro* studies indicate that GCs inhibit repair of airway epithelial cells. GCs, which are involved in a variety of physiological processes, have proved highly effective in gaining a therapeutic response from most asthmatics, by suppressing inflammation and relieving or preventing symptoms [27], [28]. Despite these effects GCs are still not able to fully reverse the damage done to airway epithelium. The failure of appropriate growth and differentiation of airway epithelial cells will cause persistent injury [29].

The repair of airway epithelium is mainly regulated through the proliferation and migration of neighboring cells around the damaged area. Currently, details of the mechanisms by which GCs inhibit the repair of airway epithelial cells remain unclear. The gene *GILZ* was originally discovered in studies aimed at characterizing genes targeted by DEX. The persistence of the *GILZ* gene and GILZ protein depends on the continuous presence of DEX, which is induced in airway epithelial cells and has an important regulatory role in the asthmatic airway [30].

Recently, detailed studies of the functions of GILZ have revealed some features for this molecule: GILZ binds to Raf-1, which is considered the most important prerequisite responsible for the inhibition of the phosphorylation of downstream Mek1/2 and Erk1/2, and inhibits the activation of the MAPK-ERK signaling pathway, which has an important role in controlling cell survival, apoptosis, proliferation, migration, and differentiation [24]. Therefore, we hypothesized that the activities of GCs that influence the repair of the airway epithelium may be mediated by GILZ.

In the current study, we investigated the expression and function of GILZ in human airway epithelial cells. Studies have shown that GCs rapidly upregulated the expression of GILZ in T lymphocytes, multiple myeloma cells, mesenchymal stem cells, and human airway epithelial cells, among others [11], [16], [30], [31]. Our results suggested that GILZ was not only upregulated by DEX but was also rapidly induced in airway epithelial cells. We also demonstrated that DEX inhibited the phosphorylation of Raf-1 and the downstream Mek1/2 and Erk1/2, and inhibited proliferation and migration. We used si-RNA technology to knockdown *GILZ* levels and investigated whether the inhibition of airway epithelial repair imposed by DEX was mediated by GILZ. Interestingly, silencing *GILZ* blocked the inhibitory effect of DEX on the MAPK-ERK signaling pathway. We also observed that the DEX-mediated decrease in proliferation and migration was due in part to the activation of GILZ in human airway epithelial cells. Altogether, our findings imply that the expression of GILZ is involved in the inhibitory effect that DEX exerts on the repair of airway epithelium. Studies have reported that the asthmatic airway epithelium showed evidence of damage, loss, and shedding to various degrees even after GCs treatment. Our results suggest that DEX, acting in part via GILZ, inhibited the repair of airway epithelium by suppressing cell proliferation and migration.

GILZ, a protein ubiquitously expressed and induced mainly by GCs, regulates the cell cycle, apoptosis, proliferation, and differentiation [32]. GILZ has an anti-inflammatory function; it modulates the activation of the MAPK-ERK signaling pathway and inhibits proliferation and migration, thereby influencing the repair of airway epithelium. GCs are widely prescribed anti-inflammatory drugs and most effective treatment available for asthma, but the negative influence on airway epithelial repair should not be ignored. In this study we found that one of the effects of GCs on the airway epithelium was the induction of GILZ expression, and that part of the reason for the inhibition of epithelial repair by GCs was the suppression of the MAPK-ERK signaling pathway and proliferation and migration. Together, these results have important implications for understanding the physiopathological role and function of GCs in the airway epithelium.
